# Use of a preclinical test in the control of classical scrapie

**DOI:** 10.1099/vir.0.022566-0

**Published:** 2010-10

**Authors:** L. A. Boden, F. Houston, H. R. Fryer, R. R. Kao

**Affiliations:** 1 Boyd Orr Centre for Population and Ecosystem Health, College of Medical, Veterinary & Life Sciences, University of Glasgow, 464 Bearsden Road, Glasgow G61 1QH, UK; 2 The Institute for Emerging Infections, The James Martin 21st Century School, Department of Zoology, Oxford University, Oxford, UK

## Abstract

Scrapie control in Great Britain (GB) was originally based on the National Scrapie Plan's Ram Genotyping scheme aimed at reducing the susceptibility of the national flock. The current official strategy to control scrapie in the national flock involves culling susceptible genotypes in individual, known affected flocks (compulsory scrapie flock scheme or CSFS). However, the recent development of preclinical test candidates means that a strategy based on disease detection may now be feasible. Here, a deterministic within-flock model was used to demonstrate that only large flocks with many home-bred ewes are likely to be a significant risk for flock-to-flock transmission of scrapie. For most other flocks, it was found that the CSFS could be replaced by a strategy using a currently available live test without excessive risk to other farmers, even if the proportion of susceptible genotypes in the flock is unusually large. Even for flocks that represent a high risk of harbouring a high prevalence of infection, there would be limited probability of onward transmission if scrapie is detected soon after disease introduction (typically less than 5 years). However, if detection of disease is delayed, the existing CSFS strategy may be the most appropriate control measure in these cases.

## INTRODUCTION

Scrapie is a naturally occurring transmissible spongiform encephalopathy (TSE) that has been endemic in the British national sheep flock for over 250 years (Parry, 2003; Radostitis *et al.*, 2000), and causes progressive neurological degeneration and death. It is associated with an abnormal form of the prion protein (PrP^Sc^) (Caughey & Chesebro, 1997).

Prioritization of scrapie eradication in Great Britain (GB) occurred after the link between bovine spongiform encephalopathy (BSE) in cattle and variant Creutzfeldt–Jakob disease (vCJD) in humans was discovered (Foster *et al.*, 1993; Bruce *et al.*, 1997; Hill *et al.*, 1997a, b). The potential for sheep to be a vCJD risk was based on four concerns: (i) sheep were demonstrated to be susceptible to BSE under experimental conditions (Foster *et al.*, 1993), (ii) they were potentially exposed to the same contaminated feed that had caused the outbreak in cattle (Kao *et al.*, 2002), (iii) the distribution of BSE-infected tissue in sheep (Jeffrey *et al.*, 2001; Somerville *et al.*, 1997) has made horizontal transmission a distinct possibility (Kao *et al.*, 2002) and (iv) as sheep affected by BSE and scrapie have very similar clinical signs and pathology, scrapie has the potential to mask an incipient BSE epidemic in sheep (Houston & Gravenor, 2003). As a result, the National Scrapie Plan (NSP) (Defra, 2007) was implemented in GB in July 2001 (http://www.defra.gov.uk/animalhealth/managing-disease/NSPAC/, discussed in Kao *et al.*, 2001). The NSP's primary objectives were to eradicate scrapie and breed for TSE resistance in the national sheep flock (http://www.defra.gov.uk/animalhealth/managing-disease/NSPAC/, discussed in Baylis *et al.*, 2002), thereby minimizing the likelihood that BSE could be present and not detected in the national flock.

The strategy was adopted due to the clear relationship between polymorphisms in the gene that encodes the prion protein and susceptibility to scrapie and BSE (Goldmann *et al.*, 1994). At the time, any strategy based purely on identifying cases of disease was considered prohibitively risky and expensive because the possible low incidence of BSE in sheep may be masked by the presence of scrapie. Thus, a genetically based breeding strategy targeting susceptibility, rather than disease, provided the most reasonable chance for success.

In 2004, the programme was augmented by a slaughter and replacement scheme. This was initially a voluntary programme, the voluntary scrapie flock scheme (VSFS) aimed at flocks known to have been scrapie-affected since 1998, but after July 2004, control became mandatory for all flocks with confirmed cases from that date, as required by EC Reg 999/2001. Upon confirmation, the compulsory scrapie flock scheme (CSFS) (Defra, 2008) requires that all sheep be slaughtered, or all susceptible sheep in the flock slaughtered and only resistant replacement animals bought in. While this has been undoubtedly effective, the relatively small incidence of scrapie means that large numbers of healthy sheep are culled.

The recent development of a live test for scrapie (González *et al.*, 2008a) suggests that preclinical testing may prove a more cost-effective disease-based strategy for scrapie eradication. Immunohistochemical staining of lymphoid biopsies from tonsil (Schreuder *et al.*, 1998) and third eyelid (O'Rourke *et al.*, 2000) have long been recognized as a method for preclinical diagnosis of TSEs. In this test, rectal biopsies are used, as anaesthesia is not required for collection, and lymphoid follicles are abundant in the rectal mucosa. Further, because the analysis can be adapted to rapid, high-throughput methods (González *et al.*, 2008b), this approach is potentially feasible for large-scale application in the field. Previous mathematical models have considered scrapie control at the flock (Stringer *et al.*, 1998; Hagenaars *et al.*, 2000; Fryer *et al.*, 2007; Truscott & Ferguson, 2009) and national level (Kao *et al.*, 2001; Gubbins, 2005; Gubbins & Webb, 2005). However, none have considered a strategy targeting infected sheep. Here, the trade-off between the number of sheep culled and the potential for onward transmission of disease after the implementation of a single (conducted at a single point in time) or sequential (conducted once each year over 3 years) live preclinical test(s) are contrasted to the CSFS. As there is strong experimental (Race *et al.*, 1998; Konold *et al.*, 2008) and epidemiological evidence (McLean *et al.*, 1999; Baylis *et al.*, 2002) that scrapie transmission is related to infected breeding sheep, the number of infected sheep likely to be sold on for breeding is used to compare between these strategies.

## RESULTS

**Fig. 1.**
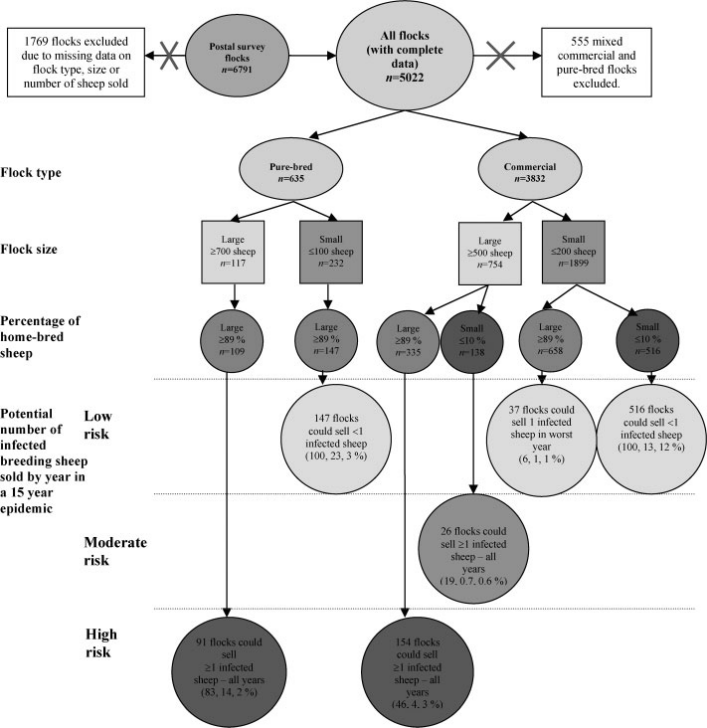
Summary of the flock parameters used in the model and description of the potential number of infected breeding sheep sold to other farms by each flock type if scrapie is present in the flock over a 15 year period. The number of flocks in each flock category reported in the postal survey data are described in each bubble. Pure-bred flocks with small proportions of home-bred sheep were not examined in this model as these flock types are extremely rare. The percentage of the flock subgroup, major flock category (commercial or pure-bred) and total flocks (commercial and pure-bred) are reported, respectively, in parentheses. For example, in the high-risk category, 83 % (91/109) of large pure-bred flocks with a large percentage of home-bred sheep sold more than one infected breeding sheep each year in a 15 year epidemic. This high-risk group comprised 14 % (91/635) of all pure-bred flocks and 2 % [91/(635+3832)] of all pure-bred and commercial flocks.

**Fig. 2.**
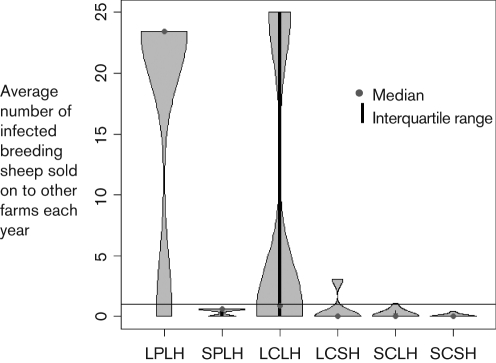
Violin plot showing the distribution of flocks with respect to the average number of infected breeding sheep sold to other farms each year by each flock type in the first year of an epidemic. The Violin plot is similar to a box plot, except that it also shows the probability density of the data at different values. The line=1 represents one infected sheep sold on per year and denotes the cut-off point between low-risk and moderate-risk flocks.

**Fig. 3.**
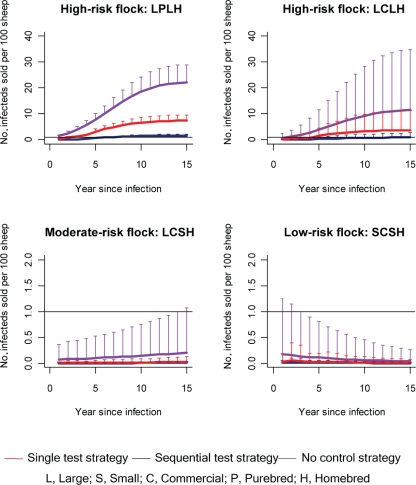
Mean number of infected breeding sheep sold by different flock types per year (in each year of an epidemic) after implementation of live-test strategies. The CSFS strategy reduced the number of infected sheep sold to zero in all cases (and is not shown on these graphs). The upper bars indicate the potential maximum number of infected breeding sheep sold per year (per 100 sheep in a flock). The minimum number of infected sheep sold per year (per 100 sheep in a flock) is zero in all years. The black line represents one infected sheep sold.

Flocks were characterized according to the risk posed to other farms. The number of infected breeding sheep sold was calculated from the number of sheep sold by each flock type and the prevalence of scrapie in the absence of a control strategy (in each year of a 15 year epidemic). A summary of each of the flock parameters and the risk they posed to other flocks is presented in Fig. 1. The mean number of infected breeding sheep sold by different flock types to other farms each year in the first year of an epidemic is described in Fig. 2. The mean number of infected breeding sheep sold per 100 sheep (in the flock) by different flock types per year in each year of a 15 year epidemic is described in Fig. 3.

### No intervention

Assuming a mean genotype distribution (39 % susceptible genotypes in the flock), over a 15 year period, scrapie prevalence decreased in small flocks (i.e. commercial flocks with ≤200 sheep and pure-bred flocks with ≤100). In these flocks, the smaller the proportion of home-bred sheep, the lower the prevalence of scrapie in the flock. In large commercial flocks (≥500 sheep), the prevalence of scrapie remained constant over a 15 year period if the proportion of home-bred sheep was small (≤0.10). However, if the proportion of home-bred sheep was large (≥0.89), scrapie prevalence in large commercial (≥500 sheep) and pure-bred flocks (≥700 sheep) increased over time.

When the proportion of susceptible genotypes in the flock was small (0.39), small pure-bred flocks with large proportions of home-bred sheep and small commercial flocks with large and small proportions of home-bred sheep sold on average less than one infected breeding sheep per year and thus were considered a low risk for onward transmission (Figs 1, 2 and 3). A small proportion of large commercial flocks with small proportions of home-bred sheep sold on ≥1 infected sheep per year (the number of infected sheep sold increased over time) (Figs 1, 2 and 3). These flocks were defined as moderate-risk flocks due to the potential for the relatively small numbers of infected breeding sheep sold on each year. The majority of large pure-bred flocks posed a high risk of onward transmission (≥1 infected sheep sold per year). A smaller proportion of large commercial flocks posed similar risks. Both of these flock types were considered to be high risk due to the relatively high potential to sell on infected breeding sheep (Figs 1, 2 and 3).

A threshold susceptible proportion was calculated for each flock type. This threshold was the proportion of susceptible genotypes in the flock which reduced the maximum risk of onward transmission to ≤1 infected sheep sold per year. In high-risk flocks, the threshold susceptible proportion was less than 5 %; in moderate-risk flocks, less than 39 % (the national average) and in low-risk flocks with large proportions of home-bred sheep, less than 50 %. In contrast, low-risk flocks with small proportions of home-bred sheep posed little risk of onward transmission even when the threshold susceptible proportion was very high (≥0.95).

### CSFS strategy (strategy 2)

While the CSFS strategy was always successful in eradicating disease, this invariably resulted in higher numbers of sheep removed and culled than the two alternative preclinical testing strategies (strategies 3 and 4), as all sheep with susceptible genotypes in the flock would be removed, regardless of disease status.

### Preclinical testing strategies (strategies 3 and 4)

There was no difference in the results between either of the testing strategies when the tests were applied to sheep at 20 or 12 months of age in any of the flock types, due to the early detection assumed for highly susceptible genotypes. However, as test sensitivity decreased, the mean number of sheep removed from the flock decreased and the risk of onward transmission increased. Even in high-risk flocks, the within-flock prevalence of scrapie was low [≤0.22 (LPLH; see Table 2)]. Therefore, both the positive predictive value (PPV) of the test (100 %) and the negative predictive value (NPV) (>97 %) were high if the test sensitivity was at least 90 %. At a similar prevalence, but with lower test sensitivities (70 and 35 %, respectively), the PPV remained unchanged, but the NPV decreased (greater than 93 and 86 %, respectively). Therefore in high-risk flocks, in the worst case scenario (with a test sensitivity of 35 %) the probability of retaining an undetected diseased sheep in the flock was 14 %. Conversely, in low-risk flocks (prevalence <1 %), with a test sensitivity of 35 %, the probability of retaining an undetected diseased sheep was less than 1 %.

Both sequential and single testing strategies were more effective at reducing prevalence and onward transmission of infection when tests were applied and assumed to be effective in sheep at 6 months of age than when testing was delayed until 12 and 20 months of age.

In all flock types, sequential-testing strategies resulted in increased numbers of infected sheep culled compared with a single-test strategy (due to the increased opportunity to detect infected sheep in subsequent years). However, sequential-testing strategies still resulted in a smaller proportion of the flock culled (percentage of flock culled in years 1–15 : 0.2–19 % in large pure-bred flocks, 0.2–0.3 % in large commercial flocks with small proportions of home-bred sheep and 0.3–11 % in large commercial flocks with large proportions of home-bred sheep) compared with the CSFS (36 % of all flocks culled).

Both live testing strategies substantially reduced the risk of onward transmission of infection in high-risk flocks (Fig. 3). The effectiveness of each strategy was dependent on when disease was detected during the course of a within-flock epidemic. At high test sensitivities (≥90 %), if scrapie was detected early in the epidemic (<5 years), sequential testing reduced the risk of onward transmission (≤1 infected breeding sheep). However, if scrapie was detected later in the epidemic (≥5 years), the majority of flocks still sold on one or more infected sheep depending on the year of implementation. A single test strategy in these high-risk flocks was not effective as the majority of flocks still sold one or more than one infected breeding sheep in the first year of the epidemic after implementation of the test. In moderate- and low-risk flocks, sequential and single testing strategies were equally effective in reducing the risk of onward transmission to other flocks (number of sheep sold <1) at any time during the epidemic.

## DISCUSSION

This study illustrates important differences in the demographics, trading patterns and ability of different flock types to sustain or perpetuate scrapie epidemics that are important for understanding the epidemic dynamics at a national level, and targeting control strategies. While this approach is invaluable for identifying broad, strategic approaches to control disease, it is not intended to provide detailed predictions about specific outbreaks.

While always successful at eliminating a within-flock epidemic of classical scrapie the CSFS strategy relies on the removal of the entire susceptible population in the flock, which potentially includes many healthy sheep. Truscott & Ferguson (2009) showed that trading restrictions alone have little power to limit transmission and a more efficient implementation could concentrate on culling or breeding restrictions; however, they did not consider the detailed demographic data from the postal surveys, which show clear distinctions amongst different flock types.

Small low-risk flocks (≤200 commercial sheep and ≤100 pure-bred sheep) were less likely to sustain an epidemic or sell on infected breeding sheep. This suggests that in these flocks, draconian control measures such as the CSFS may be unnecessary in preventing the onward spread of disease. For smaller flocks, particularly with few home-bred animals, there is both little opportunity for transmission and few susceptibles, especially with replacement of clinical cases with resistant stock. Thus, the number of susceptible animals will probably rapidly decline below a density threshold that is necessary to support endemic infection (Woolhouse *et al.*, 1998).

Similarly, the CSFS may be unnecessary for those flocks classified in this study as ‘moderate-risk flocks’. These flocks would typically be mule or other cross-bred sheep (M. Dawson, personal communication) and virtually all production would be finished lamb. Ewes would normally be culled at the end of their breeding life and as such, sheep sold on from these flocks are unlikely to be breeding sheep. Therefore, the risk for disease transmission from the sale of sheep from these flocks may be smaller than estimated in this study.

Conversely, large flocks (≥500 commercial sheep and ≥700 pure-bred sheep), with large proportions of home-bred sheep (which retained all their infected lambs), posed the greatest risk to other farms due to the increased opportunity for an epidemic to take hold. In these flocks, live preclinical testing dramatically reduced the number of sheep culled compared with the CSFS. However, testing strategies are unlikely to eradicate scrapie completely within a scrapie-positive flock due to the imperfect test sensitivity and probability of retaining infected sheep in the flock through misclassification. Even intermittent onward sale may result in epidemic persistence, if, for example, larger flocks were found to preferentially buy and sell from each other, either deliberately or inadvertently. Thus, the reduction in cost to the farmer must be balanced against the risk posed to other flocks at a national level, in particular if the test sensitivity is low in preclinical animals or if the test is too costly or difficult to implement or interpret. The onward risk of transmission by these flocks depends on the combination of having large numbers of animals at risk (Healy *et al.*, 2004; Hopp *et al.*, 2001), large numbers of replacement animals providing a steady supply of new susceptibles, and large numbers of sheep sold on to other farms. These factors therefore identify likely scenarios under which stringent control measures may be necessary and in these high-risk flocks, the existing CSFS strategy may remain the most appropriate method of control and eradication.

Use of a single test strategy may be cheaper and easier to implement. However, this strategy may result in the unacceptable exposure of additional flocks via the sale of breeding sheep from high-risk flocks. Sequential-testing strategies resulted in increased numbers of infected sheep identified and culled and are thus likely to be more expensive compared with a single-test strategy. However, as retesting in subsequent years was performed using the same rectal biopsy test, the improvement in test sensitivity from using this approach is not as great as predicted if the tests were conditionally independent (Dohoo *et al.*, 2003).

In this study, the predicted prevalence of within-flock scrapie infection in high-risk pure-bred and commercial flocks is higher than the estimated within-flock prevalence reported by Ortiz-Pelaez & Del Rio Vilas (2009) and Gubbins (2005). This may be due to differences with respect to the stage of detection of the within-flock epidemic, ability to detect disease, or most likely, the effect of stratification of flock types according to size and proportion of home-bred sheep. Nevertheless, a higher estimate of prevalence in this study (compared with others) highlights the fact that only a small proportion of the flocks in GB (high-risk pure-bred and commercial flocks) pose the greatest risk for onward transmission of scrapie infection and these should be identified and targeted in future control strategies (Fig. 1).

Future work may investigate different live-testing strategies such as testing specific genotypes only or random sampling of sheep within a flock to determine if this has an impact on the efficiency and cost effectiveness of the diagnostic test. Further information to refine flock parameters (such as different genotypic distributions for different flock types) would also assist in the identification of high-risk flocks. The logistics of testing both at a flock and a national scale must also be considered, and additional information is still required to determine the cost and ease of test implementation, the cost of culling, restrictions in breeding and trading to the farmer at an individual and national level, before further economic evaluation and comparison with the CSFS can be made.

In practice, the high-risk classification used in this study may not pertain to all large pure-bred or commercial flocks. As an example, the majority of hill breed flocks do not sell pure-bred females, although some flocks will buy in breeding ewes during particularly poor years. Typically, the best ewe lambs are retained and the remainder are finished or sold as stores. In a minority of such flocks, some ewes are ‘drafted’ to lower ground to produce mule or half-bred ewe lambs, which are then sold on to other flocks. These are the progeny of older hill ewes and may not present the same risk of carrying scrapie as the progeny of younger ewes (M. Dawson, personal communication).

This study has shown that large flocks with many home-bred breeding sheep are likely to be a disproportionate risk for onward transmission. While interpretation of the absolute risk associated with particular flock types must be viewed with caution for all the reasons stated above, the marked difference in the relative risk, due to potential for persistent within-flock transmission and onward transmission via trade suggests that scrapie could be controlled by concentrating on these few, ‘high-risk’ flocks, obviating the need for radical control measures for most flocks. In order to determine the role that these flocks might play in the persistence and spread of scrapie in the British national flock, existing datasets characterizing the detailed flock-to-flock movement of sheep in GB (Kao *et al.*, 2007; Green *et al.*, 2007; Webb, 2005) will have to be refined to consider only those movements that are important for scrapie transmission, most likely those movements involving breeding ewes. This will be the subject of future work.

## METHODS

### Within-flock spread of infectivity.

A difference equation model adapted from previous analyses (Kao *et al.*, 2002; Fryer *et al.*, 2007) was used to describe the within-flock spread of scrapie, incorporating age, infection and breeding structure, and the inheritance and impact of the PrP gene. The model takes into account flock size, the number of breeding ewes, proportion of ewes that are home-bred, proportion of ewes sold as replacements, and the genotype distribution of the flock. A full description of the model variables, parameters and equations is presented in Fryer *et al.* (2007). Model outputs were compared to determine the impact of the different control and eradication strategies on different flock structures (Supplementary Material, available in JGV Online).

### Flock parameters.

In this model, flocks were characterized according to the breeding and commercial structure of the UK industry and the role of different flock types in onward transmission of disease. These characteristics were derived from data collected in the 2002 and 2006 anonymous postal surveys (see Sivam *et al.*, 2006), which describe the demographics of British sheep flocks with and without scrapie.

Flock parameters selected for the model were based on flock type (pure-bred, commercial or mixed), flock size (25th and 75th percentiles), the proportion of home-bred sheep (25th and 75th percentiles), the likelihood of selling sheep on to other flocks and whether scrapie has ever been identified in the flock. Further information on flock parameter selection is presented in Supplementary Material.

Ultimately, there were six different flock structures defined by the number of sheep in the flock and the proportion of home-bred sheep (Fig. 1, Supplementary Material).

In the postal survey data, of flocks that have ever had scrapie, commercial flocks represented the majority (68 %). Pure-bred flocks (14.1 %) formed smaller proportions of the GB flock demographics (Sivam *et al.*, 2006). Pure-bred flocks were highly skewed with respect to the distribution of flock size (mean 366, median 153, range 9–3270) and proportion of home-bred sheep (mean 0.85, median 0.98, range 0–1.0). Similarly, commercial flocks were skewed with respect to flock size (mean 331, median 204, range 13–5512), but normally distributed with respect to the proportion of home-bred sheep (mean 0.58, median 0.53, range 0–1.0).

Consistent with the postal survey data, it was assumed that 98 % of the breeding sheep within each flock are ewes and the remaining 2 %, rams. Although some pure-bred flocks will breed all their own replacements, in this model, all flocks were assumed to sell ewes as replacements.

### Genotype distribution.

An average genotype distribution (equal to the national distribution) was used to determine the impact of different control strategies on the different flock types with respect to the number of sheep culled and infected sheep sold on to other farms. This was based on the prevalence of genotypes described in abattoir active surveillance data (VLA 2003a, b). There were six genotypes described (in order of increasing susceptibility to infection): ARR/ARR, ARR/AXX, AXX/AXX, ARR/VRQ, AXX/VRQ and VRQ/VRQ. The AXX genotype refers to ARQ, AHQ and ARH genotypes. Genotype properties are described in Table 1 (see also Fryer *et al.*, 2007).

In order to consider the impact of flocks with unusually large proportions of susceptible genotypes (i.e. not ARR/ARR or ARR/AXX), the number of infected sheep sold on to other farms was also compared for different flock types with different genotype distributions. We calculated a threshold susceptible proportion for each flock type. This threshold was the proportion of susceptible genotypes in the flock which reduced the maximum risk of onward transmission to ≤1 infected sheep sold per year. We examined a range of proportions of susceptible genotypes in each flock type (0, 0.05, 0.1, 0.2, 0.3, 0.4, 0.5, 0.6, 0.7, 0.8, 0.9 and 0.95). The relative distribution of each of the six individual genotypes within each of these scenarios was derived using the ratios of each genotype in the abattoir screening data (Table 1).

### Control and eradication strategies.

The model was adapted to consider the impact of three proposed control and eradication strategies for scrapie and contrasted with a strategy where no intervention occurs for up to 15 years (the estimated average duration of a within-flock epidemic) (Gravenor *et al.*, 2001). In the model, it was assumed that the presence of an index case has identified an ongoing scrapie epidemic, defined as a holding that has at least one case of home-bred scrapie in the last year or at least two cases of scrapie in the last 5 years, of which at least one was home-bred (Fryer *et al.*, 2007). The model assumed that most sheep get infected within the first few months of life but that no sheep under the age of 12 months die from scrapie (Fryer *et al.*, 2007). The model runs for a period of 5 years after the year of strategy implementation to consider the short-term impact of the differing strategies, assuming that the year of detection occurred anywhere between 1 and 15 years after introduction of at least one infected sheep. This was done to take into account preclinical infection that may otherwise go undetected due to the long incubation period of the disease.

#### Strategy 1.

In this strategy, no intervention was implemented. In these flocks, no scrapie-positive sheep were culled or removed from the flock at any point in time during the epidemic.

#### Strategy 2.

This strategy was modelled on the current CSFS (http://www.defra.gov.uk/animalhealth/publications/NSPAC/). All sheep are tested for genotype. This is a reactive policy enforced in response to detected disease in the flock. Susceptible genotypes were culled and resistant genotypes (ARR/ARR ewes and rams and ARR/AXX ewes) retained (Defra, 2007). For 2 years after the first year of implementation of this strategy, only resistant sheep were bought in (ARR/ARR ewes and rams and ARR/AXX ewes). After 2 years of restrictions, replacement sheep were bought in, in proportion to the distribution of the genotype of the national flock.

#### Strategy 3.

A preclinical diagnostic test was conducted each year over three consecutive years. A 3 year testing strategy was chosen initially to reflect similar time and trade restrictions imposed by the CSFS. Like the CSFS, for 2 years after the first year of implementation of this strategy, the model restricted the genotype of bought in sheep to those genotypes resistant to scrapie (ARR/ARR ewes and rams and ARR/AXX ewes). After 2 years of restrictions, replacement sheep were bought in, in proportion to the distribution of the genotype of the national flock.

#### Strategy 4.

A preclinical diagnostic test was conducted once (at a single time within a single year with subsequent restrictions enforced on sale, purchase and breeding on the holding for a period of 3 years), otherwise as for strategy 3.

### Live test parameters.

The live preclinical test implemented in strategies 3 and 4 was based on the experimental support for rectal biopsies of lymphoid tissue and is described in detail by González *et al.* (2008a). This test is not currently a pen-side test. Under experimental conditions, when the prevalence of disease was 100 %, González *et al.* (2008a) found that the risk of a false-negative result in a preclinical rectal biopsy sample was 9.3 % if the sample contained 10 follicles. The probability of obtaining a sample containing at least 10 follicles was 87 %. The risk of a false-positive result was believed to be negligible (L. González, personal communication).

For this model, it was assumed that every sheep in the flock (over a specified age) was tested and that at least 10 follicles were retrieved in every single biopsy sample. An initial individual test sensitivity of 90 % and specificity of 100 % were chosen for the model. Lower individual test sensitivities (<90 %) were also investigated. Specificity was assumed to be 100 % in each scenario. PPV (the probability that given a positive test result, the sheep has the disease) and NPV (the probability that given a negative test result, the sheep does not have the disease) were calculated for different flock structure prevalences using different test sensitivities (90, 70 and 35 %).

In the model, the live preclinical testing strategies were applied to sheep of different ages (greater than 20, 12 and 6 months). These ages were based on data from González *et al.* (2008a) which suggest that the first positive tests in the rectal mucosa appear at statistically similar average proportions of the incubation period of experimentally infected sheep (0.5 in AXX/AXX sheep, 0.49 in AXX/VRQ and 0.43 in VRQ/VRQ sheep). Although the chances of a positive test result increase with age, infected VRQ/VRQ sheep can be detected as early as 4–8 months post-infection; ARQ/VRQ and ARQ/ARQ sheep can be detected as early as 8 months post-infection. Most of these genotypes are detected at 16 months post-infection; ARR/VRQ sheep are detected as early as 24 months post-infection but most are detected at 36–48 months post-infection) (L. Gonzalez, personal communication)

### Model outputs.

The mean number of breeding sheep culled due to scrapie and the mean prevalence of scrapie per year were calculated for each flock type and year past the initial intervention. In addition, data from the scrapie postal survey (2002) on all flocks were used to estimate the potential number of infected breeding sheep sold to other farms after each intervention for each flock type is investigated. A qualitative comparison of the number of sheep sold by each flock type indicates that there is a negligible difference between scrapie-positive flocks and scrapie-negative flocks, so data from all flocks were used. The number of sheep sold was recorded categorically in the scrapie postal survey (0, 1–5, 6–20, 21–50, 50–100 and greater than 100 sheep sold). In order to obtain the maximum number of breeding sheep sold on from an infected flock per annum, categorical data were interpreted in terms of the upper bounds of each category (i.e. 0, 5, 20, 50, 100 and >100 sheep). An estimate of the upper bounds of the final category (>100 sheep) was based on the average size of the breeding ewe flock in each flock type (in the final category), assuming that each breeding ewe produces at least one ewe lamb per year (Table 2). This is an upper estimate for hill flocks, as most pure-bred ewes produce an average of 0.5–0.75 ewe lambs per year (M. Dawson, personal communication). The average prevalence of scrapie in the flock, after each strategy was implemented, was used to calculate the number of infected sheep sold on subsequently to other flocks each year for each flock type (Table 2). For each flock type, the mean number of infected breeding sheep sold onwards per flock per year was calculated for years 1–15 of an epidemic. The mean number of infected breeding sheep sold on was divided by the flock size for each flock type to obtain a per capita onward risk so that direct comparisons could be made between different flock types. For each flock type, this output was multiplied by 100 to obtain the number of infected breeding sheep sold per 100 sheep in the flock. Model outputs were compared to determine the impact of the different control and eradication strategies on different flock structures.

## Supplementary Material

[Supplementary Material]

## Figures and Tables

**Table 1.** Average sheep genotypes: prevalence, relative susceptibility and mean age at onset of scrapie (reproduced from Fryer *et al.*, 2007) na, Not applicable.

**Genotype**	**Prevalence in abattoir screening – national genotype (%)**	**Estimated relative susceptibility to scrapie**	**Mean age of onset of scrapie symptoms (years)**
ARR/ARR	19.5	0	na
ARR/AXX	41.9	0.001	5.0
AXX/AXX	26.5	0.026	3.8
ARR/VRQ	5.5	0.119	5.9
AXX/VRQ	6.2	0.359	3.8
VRQ/VRQ	0.4	1.000	3.2

**Table 2.** Prevalence and proportion of flocks within each flock type selling on sheep to other farms L, Large; S, small; P, pure-bred; C, commercial; H, home-bred.

**Flock type**	**No. flocks in postal survey (% of all study flocks *n*=5022)**	**Prevalence (range years 1–15)**	**Proportion (and number) of each flock type selling sheep to other farms (by category of sheep sold)**						**Estimated maximum number of sheep sold (in >100 category)**
			**0 sheep**	**5 sheep**	**20 sheep**	**50 sheep**	**100 sheep**	**>100 sheep**	
**Low-risk flocks**									
SPLH	147 (3 %)	1.0–0.2 %	0.32 (47)	0.05 (8)	0 (0)	0.63 (92)	0 (0)	0 (0)	50
SCLH	37 (1 %)	1.0–0.2 %	0.71 (466)	0.02 (10)	0.09 (59)	0.13 (86)	0.04 (28)	0.01 (9)	103
SCSH	516 (12 %)	0.4–<0.001 %	0.83 (429)	0.004 (2)	0.03 (14)	0.05 (25)	0.04 (23)	0.04 (23)	100
**Moderate-risk flocks**									
LCSH	26 (0.6 %)	0.3–0.9 %	0.73 (103)	0 (0)	0.01 (2)	0.01 (1)	0.04 (6)	0.19 (27)	993
**High-risk flocks**									
LPLH	91 (2 %)	1–22 %	0.10 (11)	0 (0)	0.02 (2)	0.05 (5)	0.08 (9)	0.75 (82)	1210
LCLH	154 (3 %)	2–27 %	0.46 (154)	0.01 (2)	0.01 (4)	0.06 (21)	0.14 (48)	0.32 (106)	1373
