# Supplementary material for: Use of a preclinical test in the control of classical scrapie
Source: J Gen Virol. 2010 Oct;91(Pt 10):2642–50. doi: 10.1099/vir.0.022566-0 (PMC3052601; doi:10.1099/vir.0.022566-0)
Supplement: [Supplementary Material] [file supp_91_10_2642__index.html]

 Use of a preclinical test in the control of classical scrapie -- Boden et al. 91 (10): 2642 Data Supplement - Supplementary Material -- Journal of General Virology

## 

### Use of a preclinical test in the control of classical scrapie, by L. A. Boden, F. Houston, H. R. Fryer and R. R. Kao

*Journal of General Virology* vol. **91**, part 10, pp. 2642 - 2650

**Supplementary Material**  [PDF]  (87 KB)

  
  
