# Supplementary material for: Use of a preclinical test in the control of classical scrapie
Source: J Gen Virol. 2010 Oct;91(Pt 10):2642–50. doi: 10.1099/vir.0.022566-0 (PMC3052601; doi:10.1099/vir.0.022566-0)
Supplement: [Supplementary Material] [file supp_91_10_2642__1.pdf]

A mathematical model of the within-flock spread of classical scrapie

Presented here is a partial reproduction of the mathematical model produced by Fryer *et al.* (2007). This model was adapted to include two testing strategies using a live preclinical test for scrapie.

Flock demography

Lambing

Within the UK, lambs are born all year round to satisfy the continuous demand for meat. However, the main lambing period is around April to May and most ewes only lamb once per year, so the model reflects this by having pulse births once per year. It is assumed that ewes are not mated until they are 2 years old (Pollott, 1998) and that the number of lambs born each year is proportional to the number of ewes over this age. The lambing rate has been chosen to closely match an estimate made in 1999 that there is, on average, 1.3 lambs raised per ewe mated (Merrell, 2000). This value has been adjusted slightly to 1.2 to give the correct proportions of breeding sheep and lambs in the population, as described by the 2003 Agricultural Census (Defra, 2003).

Flock size and sale and purchase of lambs

In the model the number of ewes in the flock can be chosen and so too can the proportion of replacements that are home-bred and the proportion of the remaining lamb crop that are sold as replacements. If replacement ewes and rams are not kept from the lamb crop they are bought in, uninfected, from other farms at the same point in time.

Age-structure of breeding sheep in the flock

This is an age-structured model and to derive estimates for the rate at which breeding sheep in the flock die and are culled for food we considered two pieces of data. The age distribution of sheep in the flock is chosen to match the age distribution of sheep in the national flock, described as a survival distribution in McLean *et al.* (1999) (see Fig. 1). We also assume that no animals live beyond 8 years of age. The ratio of sheep that die to those that are culled for food has been chosen to give the correct proportion of lambs and breeding sheep slaughtered each year (in 2003 around 3.01 million lambs and 0.44 million breeding sheep were slaughtered) (Defra, 2002).

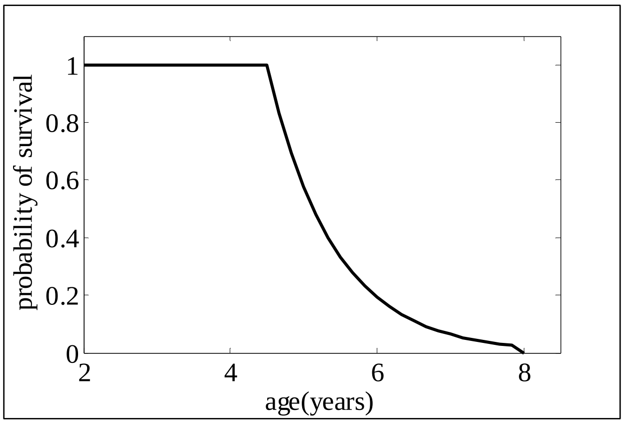

Fig. 1. Estimated survival distribution of breeding sheep. Data from McLean *et al.* (1999).

Modelling genetics in the flock

There are three TSE-related alleles that have been identified – ARR, AXX and VRQ. The AXX genotype refers to ARQ, AHQ and ARH genotypes. Each lamb will have a pair of alleles; one coming from each parent. In this model the proportion of offspring of each genotype will be determined by the proportion of each of the alleles in the male population and each of the alleles in the female population, as described by Mendelian genetics.

Flock types

In the UK, flock classifications are based on breed and the role they play in the industry (Truscott & Ferguson, 2009). There is also a recognized flow of animals, genetic material and hence infection based on grazing altitude (hill to lowland) as well as from pure-bred to cross-breeding flocks (Pollott & Stone, 2003; Truscott & Ferguson, 2009).

Flock parameters selected for the model are based on flock type (pure-bred, commercial or mixed), flock size (25th and 75th percentiles), the proportion of home-bred sheep (25th and 75th percentiles), the likelihood of selling sheep on to other flocks and whether scrapie has ever been identified in the flock. Due to the difference in the distribution of the number of sheep sold, pure-bred and commercial flocks, as defined by the correlation between flock size and flock type, were the major flock categories retained in the model. As the numbers of sheep in mixed commercial and pure-bred scrapie-positive flocks are similar to both commercial and pure-bred scrapie-positive flock types, this mixed flock type was not investigated further.

Further stratification of flock types into small and large commercial, pure-bred and mixed hill, upland and lowland flocks was also investigated. According to the postal survey data, farm type (hill, upland and lowland flocks) appears to be correlated with flock type. Independently, farm type does not appear to play a significant role in the risk of being scrapie positive and does not have an impact on the number of sheep sold on to other flocks (Hoinville *et al.*, 2000). Therefore these subclassifications were not used.

Ultimately, there were six different flock structures defined by the number of sheep in the flock and the proportion of home-bred sheep. Flock size and proportion of home-bred sheep were based on the 25th and 75th percentiles. Pure-bred flocks were highly skewed with respect to the distribution of flock size (mean 366, median 153, range 9–3270) and proportion of home-bred sheep (mean 0.85, median 0.98, range 0–1.0). Similarly, commercial flocks were skewed with respect to flock size (mean 331, median 204, range 13-5512) but normally distributed with respect to the proportion of home-bred sheep (mean 0.58, median 0.53, range 0–1.0).

Specifically the six flock structures were:

- Large pure-bred flocks (≥700 sheep) with large proportions of home-bred sheep (≥0.89)
- Small pure-bred flocks (≤100 sheep) with large proportions of home-bred sheep (≥0.89)
- Large commercial flocks (≥500 sheep) with large proportions of home-bred sheep (≥0.89)
- Large commercial flocks (≥500 sheep) with small proportions of home-bred sheep(≤0.10)
- Small commercial flocks (≤200 sheep) with large proportions of home-bred sheep (≥0.89)
- Small commercial flocks (≤200 sheep) with small proportions of home-bred sheep(≤0.10)

There were few large (*n*=4 flocks) or small (*n*=19 flocks) pure-bred flocks with small proportions of home-bred sheep (≤0.10) reported in the postal survey data so these flocks were not included in further analyses. Moderate sized pure-bred and commercial flocks (with numbers of sheep between the 25th and 75th percentiles) were also examined to determine their impact on the selling on of infected sheep. Moderate sized pure-bred flocks (mean size 290, median 226, range 102–685, mean proportion of home-bred sheep 0.85, median proportion of home-bred sheep 0.98) had similar trading behaviour as flocks classified as small pure-bred flocks with large proportions of home-bred sheep and therefore were not considered further within the model. Moderate sized commercial flocks (mean size 318, median size 308, range 203–499, mean proportion of home-bred sheep 0.48, median proportion of home-bred sheep 0.46) were not considered further due to their similarity to the small commercial flocks.

In the postal survey data, of flocks that have ever had scrapie, commercial flocks represent the majority (68%). Pure-bred flocks (14.1%) and mixed flocks (17.5%) formed smaller proportions of the GB flock demographics (Sivam *et al.*, 2006).

Within-flock spread of classical scrapie

Susceptibility to classical scrapie

Under natural conditions, a sheep's genotype is a major influence on its susceptibility to a scrapie infection (Table 1).

Table 1. Average sheep genotypes: prevalence and susceptibility (reproduced from Fryer *et al.*, 2007)

Genotype	Prevalence in abattoir screening - national genotype (%)	Estimated susceptibility to scrapie
ARR/ARR	19.5	0
ARR/AXX	41.9	0.001
AXX/AXX	26.5	0.026
ARR/VRQ	5.5	0.119
AXX/VRQ	6.2	0.359
VRQ/VRQ	0.4	1.000

Incubation periods for scrapie

Mean incubation periods for scrapie are presented in Table 2.

Table 2. Incubation periods for scrapie

Genotype	Incubation period (years)		
	Scrapie (IC)	Scrapie (oral)	Scrapie (natural*)
ARR/ARR			
ARR/AXX			5.0
AXX/AXX	2.5	5.0	3.8
ARR/VRQ	1.8	4.2	5.9
AXX/VRQ		3.2	3.8
VRQ/VRQ			3.2

*The age of reported scrapie cases (data from VLA) is used for the incubation period of natural scrapie. This is consistent with the model's assumption that most sheep get infected in the first few months of life.

Horizontal transmission

It is clear that scrapie can be transmitted horizontally (from sheep to sheep through direct or indirect contact) under natural conditions.

Here, the likelihood that a sheep will become infected is determined by two things: firstly how susceptible that sheep is, and secondly the total burden of infectiousness of the flock at that time. Infectiousness of the flock is dependent upon the number of infected sheep in the flock and upon the level of infectivity that has accumulated within each infected sheep.

Total infectivity in a sheep over the course of an infection is estimated by summing over the infectivity present in each tissue in the sheep (Table 3). Total infectivity per sheep, scaled to 1 at the clinical stage, is used to define how infectious sheep of different genotypes are over the course of an infection (Fig. 2). This is calculated by assuming that each sheep reaches the limit of infectiousness by the end of its incubation period.

Table 3. Total TSE-infectivity present in a sheep over the course of an infection

Time since infection	3 months	6 months	11 months	25 months	36 months (clinical stage)
Total infectivity in sheep (millions mouse intracerebral ID50s)	0.022	0.93	26	74	1200
(Infectivity)/(infectivity at clinical stage)	0.00002	0.0008	0.02	0.06	1

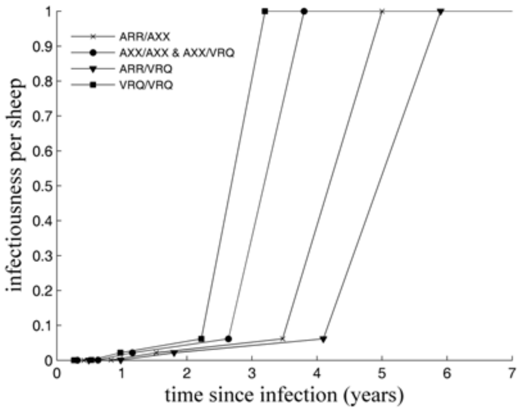

Fig. 2. Normalized infectivity of a sheep as a function of time since infection, for each genotype. The total infectiousness of the flock is the estimated at any time point as the sum of each sheep's infectiousness. This value is then scaled to give within-flock epidemics of comparable sizes to natural scrapie epidemics (Fig. 3). We assume that a flock with around 1070 breeding ewes that breeds all its own replacements has around 10 clinical cases 10 years into an epidemic.

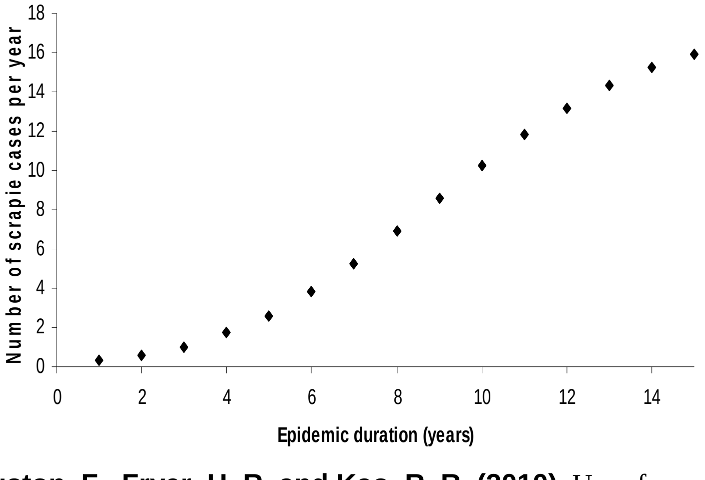

Boden, L. A., Houston, F., Fryer, H. R. and Kao, R. R. (2010). Use of a preclinical test in the control of classical scrapie. *J Gen Virol* **91**, 2642–2650.

Fig. 3. Number of clinical scrapie cases per year in flocks that breed over 98% of their replacements and have over 1070 breeding ewes. The epidemic duration represents the time that the farmer found the first suspect case of clinical scrapie on the farm. Data from Institute for Animal Health (IAH) scrapie flock study 2002.

Vertical transmission

The model also accounts for the possibility of vertical transmission from ewe to lamb. It is assumed that the probability of transmission is dependent upon the genotype of the lamb and the infection stage of the ewe. The probability of infection increases as the infection stage of the ewe increases and is bounded by the value of relative susceptibility corresponding to the lambs' genotype, as described in section 2.1.

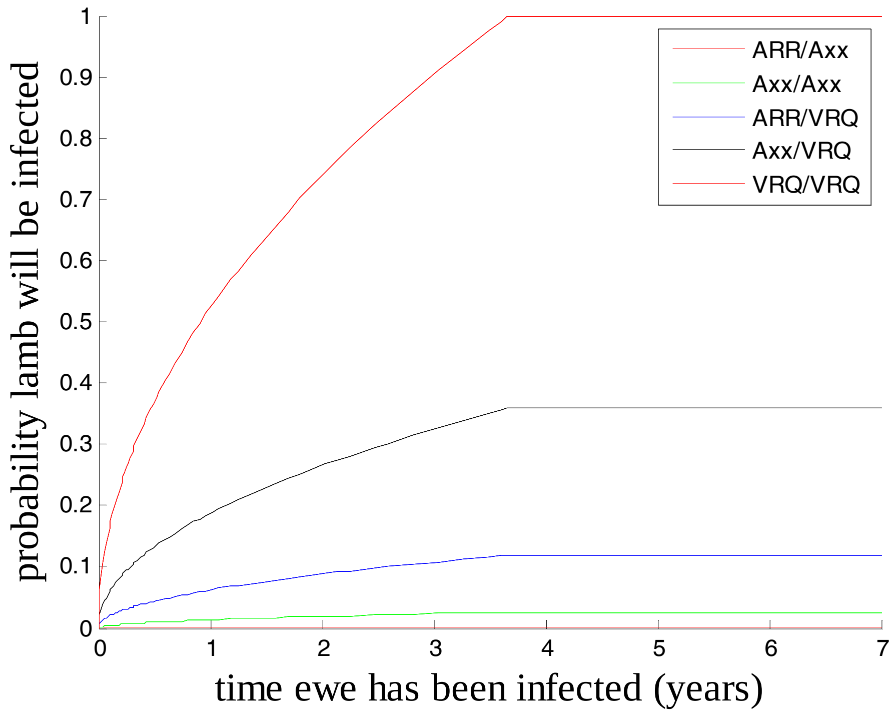

Fig. 4. A plot showing the assumed probability of vertical transmission for lambs of different genotypes as a function of how long the ewe has been infected.

The effectiveness of disease-specific PrP tests

To estimate the overall effectiveness of a control strategy that involves disease-specific PrP testing requires an estimation of how reliable these tests are at detecting positive scrapie samples.

Live test parameters

The live preclinical test is based on the experimental support for rectal biopsies of lymphoid tissue and is described in detail by González *et al.* (2008). They found that the risk of a false-negative result in a preclinical rectal biopsy sample was 9.3% if the sample contained 10 follicles and that the probability of obtaining a sample containing at least 10 follicles was 87%. The risk of a false positive is believed to be negligible (L. González, personal communication) Frequency of detection of the abnormal prion protein (PrP^d) was only slightly higher in samples of palatine tonsil and retropharyngeal lymph node of infected sheep, but these tissues are much less accessible than the rectal mucosa and therefore considered unsuitable for large scale application of a live animal test (González *et al.*, 2006). For this model, it was assumed that at least 10 follicles are retrieved in a single biopsy sample and accordingly, initial test sensitivity of 90% and specificity of 100% was chosen for the model. Test sensitivities of 70% and 35% are also investigated.

In the model, the live preclinical testing strategies are applied to sheep of different ages (greater than 20, 12 and 6 months). These ages were based on data from González *et al.* (2008) which suggest that the first positive tests in the rectal mucosa appear at statistically similar average proportions of the incubation period of experimentally infected sheep (0.5 in AXX/AXX sheep, 0.49 in AXX/VRQ and 0.43 in VRQ/VRQ sheep). Although the chances of a positive test result increase with age, infected VRQ/VRQ sheep can be detected as early as 4–8 months post-infection; VRQ/ARQ and ARQ/ARQ sheep can be detected as early as 8 months post-infection and substantially at 16 months post-infection; VRQ/ARR sheep are detected as early as 24 months post-infection but most are detected at 36-48 months post-infection) (L. Gonzalez, personal communication)

Variables and parameters (Fryer *et al.*, 2007)

Independent variables

t = time (each timestep represents 2 months)

i = age (each timestep represents 2 months)

j = time infected (each timestep represents 2 months)

s = sex (1=female, 2=male)

g = genotype (1=ARR/ARR, 2=ARR/AXX, 3=AXX,/AXX,, 4=ARR/VRQ, 5=AXX,/VRQ, 6=VRQ/VRQ)

State variables

$X_{g,i,s}(t) =$ number of susceptible sheep of genotype g, age i and sex s at time t

$Y_{g,i,j,s}(t) =$ number of infected sheep of genotype g, age i, infected for time j and sex s at time t

Parameters

$L_{g,j}$ = normalised infectivity of a sheep of genotype g, infected for time j

τ_g = mean incubation period of sheep of genotype g

$\mu_{i,s}^{die}$ = rate at which sheep aged i and sex s die

$\nu_{g,j}$ = probability that a lamb of genotype g born to a ewe of infecion stage j will be infected at birth

a_g = shape of gamma distribution of incubation periods for genotype g

b_g = scale of gamma distribution of incubation periods for genotype g

η_g = relative susceptibility of sheep of genotype g

A_i = relative susceptibility of a sheep aged i

p_s^{hbred} = proportion of breeding sheep of sex s, that are homebred

$p_s^{replace}$ = proportion of the lambs of sex s, not kept as home replacements, that are sold as replacements

ϖ_{g} = proportion of sheep that are of genotype g in the flock initially

γ = population birth rate

$\alpha_{g,j}$ = disease related death/cull rate of infected sheep of genotype g at infection stage j

σ = age at which ewes start to reproduce

$\beta_{g,i}$ = susceptibility of a susceptible sheep of genotype g and age i

β = susceptibility constant

N_{fem} = the no. of females, aged 4 timesteps and over, in the 4th time step of every year (predetermined constant)

Auxiliary variables

$B_g(t)$ = proportion of all births that are susceptible sheep of genotype g

$\tilde{B}_g(t)$ = proportion of all births that are infected sheep of genotype g

$\lambda_{g,i}(t)$ = force of infection for susceptible sheep of genotype g and age i

$N_s^{breed}(t)$ = total number of breeding sheep of sex s in population (age σ and over)

Equations

Flock demography and within-flock transmission

$$X_{g,1,s}(t+1)=\begin{cases}\dfrac{B_g(t)}{2}\gamma N_1^{breed}(t) & t=6n,\ n=0,1,2,..\\ 0 & \text{otherwise}\end{cases}$$

$$X_{g,4,s}(t+1)=\begin{cases}\left(1-p_s^{hbred}\right)\pi_s(t)\varpi_g+\left(1-\mu_{3,s}(t)\right)\left(1-\lambda_{g,3}(t)\right)X_{g,3,s}(t) & t=6n+3,\ n=0,1,2,..\\ 0 & \text{otherwise}\end{cases}$$

$$X_{g,i,s}(t+1)=\left(1-\mu_{i-1,s}(t)\right)\left(1-\lambda_{g,i-1}(t)\right)X_{g,i-1,s}(t) \qquad i=2,3\,\&\,5,...,50$$

$$Y_{g,1,1,s}(t+1)=\begin{cases}\dfrac{\tilde{B}_g(t)}{2}\gamma N_1^{breed}(t) & t=6n,\ n=0,1,2,...\\ 0 & \text{otherwise}\end{cases}$$

$$Y_{g,i,1,s}(t+1)=\lambda_{g,i-1}(t)\left(1-\mu_{i-1,s}(t)\right)X_{g,i-1,s}(t) \qquad i=2,3,...,50$$

$$Y_{g,i,j,s}(t+1)=\left(1-\min\left(\mu_{i-1,s}(t)+\alpha_{g,j-1},1\right)\right)Y_{g,i-1,j-1,s}(t) \qquad i=2,3,...,50,\ \ j=2,3,...,i$$

$$N_s^{breed}(t)=\sum_{g=1}^{6}\sum_{i=\sigma}^{50}\left(X_{g,i,s}(t)+\sum_{j=1}^{i}Y_{g,i,j,s}(t)\right)$$

$$\mu_{i,s}(t)=\mu_{i,s}^{food}(t)+\mu_{i,s}^{sell}(t)+\mu_{i,s}^{die}(t)$$

$$\lambda_{g,i}(t)=\min\left(\beta\eta_g A_i\sum_{g=1}^{6}\sum_{i=1}^{50}\sum_{j=1}^{i}L_{g,j}\sum_{s=1}^{2}Y_{g,i,j,s}(t),1\right)$$

$$\pi_s(t)=N_{fem}+\sum_{g=1}^{6}\left(\sum_{\substack{i=1\\ i\neq 3}}^{50}\left(\mu_{i,1}X_{g,i,s}(t)+\sum_{j=1}^{i}\min\left(\mu_{i,1}(t)+\alpha_{g,j},1\right)Y_{g,i,j,s}(t)\right)-\sum_{i=4}^{50}\left(X_{g,i,s}(t)+\sum_{j=1}^{i}Y_{g,i,j,s}(t)\right)\right)$$

$$\varepsilon_s(t)=p_s^{hbred}\pi_s(t)\Big/\sum_{g=1}^{6}\left(X_{g,3,s}(t)+\sum_{j=1}^{i}Y_{g,3,j,s}(t)\right)$$

$$\mu_{i,s}^{food}(t)=\begin{cases}0 & i=1,2\ \&\ 4,5,...,27\\ 0.092 & i=28,29,...,47\\ 0.5416 & i=48,49,50\\ \left(1-p_s^{replace}\right)\left(1-\varepsilon(t)\right) & i=3,\ t=3+6n\ (n=0,1,2,..)\\ 0 & i=3,\ t\neq 3+6n\ (n=0,1,2,..)\end{cases}$$

$$\mu_{i,s}^{sell}(t)=\begin{cases}0 & i=1,2\ \&\ 4,5,...,50\\ p_s^{replace}\left(1-\varepsilon_s(t)\right) & i=3,\ t=3+6n\ (n=0,1,2,..)\\ 0 & i=3,\ t\neq 3+6n\ (n=0,1,2,..)\end{cases}$$

Mendelian genetics and vertical transmission

$$\nu_{g,j}=\eta_g\times\min\left(\left(j/22\right)^{\frac{1}{2}},1\right)$$

$$\rho_{ARR,g}(t)=\begin{cases}\left(\sum_{i=\sigma}^{50}\left(X_{1,i,1}(t)+\frac{1}{2}X_{2,i,1}(t)+\frac{1}{2}X_{4,i,1}(t)+\sum_{j=1}^{i}\left(1-\nu_{g,j}\right)\left(Y_{1,i,j,1}(t)+\frac{1}{2}Y_{2,i,j,1}(t)+\frac{1}{2}Y_{4,i,j,1}(t)\right)\right)\right)\Big/N_1^{breed}(t) & g=1,2,4\\ 0 & g=3,5,6\end{cases}$$

$$\tilde{\rho}_{ARR,g}(t)=\begin{cases}\left(\sum_{i=\sigma}^{50}\sum_{j=1}^{i}\nu_{g,j}\left(Y_{1,i,j,1}(t)+\frac{1}{2}Y_{2,i,j,1}(t)+\frac{1}{2}Y_{4,i,j,1}(t)\right)\right)\Big/N_1^{breed}(t) & g=1,2,4\\ 0 & g=3,5,6\end{cases}$$

Similarly for $\rho_{Axx,g}(t)$, $\tilde{\rho}_{Axx,g}(t)$, $\rho_{VRQ,g}(t)$ and $\tilde{\rho}_{VRQ,g}(t)$

Boden, L. A., Houston, F., Fryer, H. R. and Kao, R. R. (2010). Use of a preclinical test in the control of classical scrapie. *J Gen Virol* **91**, 2642–2650.

$$z_{ARR}(t) = \left(\sum_{i=\sigma}^{50} \left(X_{1,i,2}(t) + \frac{1}{2} X_{2,i,2}(t) + \frac{1}{2} X_{4,i,2}(t) + \sum_{j=1}^{i} \left(Y_{1,i,j,2}(t) + \frac{1}{2} Y_{2,i,j,2}(t) + \frac{1}{2} Y_{4,i,j,2}(t) \right) \right) \right) \Big/ N_2^{breed}(t)$$

Similarly for $z_{Axx}(t)$ and $z_{VRQ}(t)$

$$B_1(t) = z_{ARR}(t)\rho_{ARR,1}(t), \qquad B_2(t) = z_{ARR}(t)\rho_{AXX,2}(t) + z_{AXX}(t)\rho_{ARR,2}(t)$$

$$\tilde{B}_1(t) = z_{ARR}(t)\tilde{\rho}_{ARR,1}(t), \qquad \tilde{B}_2(t) = z_{ARR}(t)\tilde{\rho}_{AXX,2}(t) + z_{AXX}(t)\tilde{\rho}_{ARR,2}(t)$$

Similarly for $g = 3,4,5,6$

Parameter estimations

$\gamma = 1.2$

$\sigma = 12$

$$\mu_{i,s}^{die} = \begin{cases} 0 & i = 1,2,...,27 \\ 0.078 & i = 28,29,...,47 \\ 0.4584 & i = 48,49,50 \end{cases}$$

$$A_i = \begin{cases} 1 & \text{i=1,2,3} \\ 0.04 & \text{otherwise} \end{cases}$$

$$(\eta_1,\ \eta_2,\ \eta_3,\ \eta_4,\ \eta_5,\ \eta_6) = (0,\ 0.001,\ 0.026,\ 0.119,\ 0.359,\ 1)$$

$$\alpha_{g,j} = \begin{cases} 0 & j = 1,2,...,6 \\ \dfrac{\int_{x=2(j-1)}^{2j} f(x, a_g, b_g)\, dx}{\int_{\hat{x}=2(j-1)}^{\infty} f(\hat{x}, a_g, b_g)\, d\hat{x}} & \text{otherwise} \end{cases}$$

where $f(x,a,b) = \dfrac{x^{a-1} e^{-\frac{x}{b}}}{b^a \int_{t=0}^{\infty} e^{-t} t^{a-1} dt}$ is the density function of a Γ(a,b) distribution

$a_g = 7.3 \quad \forall g$

$(b_2, b_3, b_4, b_5, b_6) = (4.1,\ 3.1,\ 4.8,\ 3.1,\ 2.6)$

$(\tau_2, \tau_3, \tau_4, \tau_5, \tau_6) = (30,\ 23,\ 35,\ 23,\ 19)$

$$\Omega_i = \begin{cases} 1 & i = 1,2,...,6 \\ (3i+20)/38 & i = 7,8,...,11 \\ 28/19 & i = 12,13,...,50 \end{cases}$$

$$(\varpi_1,\ \varpi_2,\ \varpi_3,\ \varpi_4,\ \varpi_5,\ \varpi_6) = (0.195,\ 0.419,\ 0.265,\ 0.055,\ 0.062,\ < 0.04)$$

References

Defra (2002). Slaughter statistics 2002. www.defra.gov.uk.

Defra (2003). *Agricultural census data 2002 and 2003: Department for Environment*. Food and Rural Affairs.

DNV (2004). Audit of sheep and goat meat, & dairy products and their uses – ZM0307: DNV & Foods Standards Agency.

Fryer, H. R., Baylis, M., Sivam, K. & McLean, A. (2007). Quantifying the risk from ovine BSE and the impact of control strategies. *Proc Biol Sci* **274**, 1497–1503. Medline

González, L., Dalgleish, M. P., Martin, S., Dexter, G., Steele, P., Finalyson, J. & Jeffrey, M. (2008). Diagnosis of preclinical scrapie in live sheep by the immunohistochemical examination of rectal biopsies. *Vet Rec* **162**, 397–403. Medline

McLean, A. R., Hoek, A., Hoinville, L. J. & Gravenor, M. B. (1999). Scrapie transmission in Britain: a recipe for a mathematical model. *Proc Biol Sci* **266**, 2531–2538. Medline

Merrell, B. G. (2000). An examination of the numbers of sheep and the sheep breeding structure in the UK.

Pollott, G. E. (1998). *Sheep breeds and breeding in Britain 1996/7. Sheep yearbook*. Meat and Livestock Commission.

Pollott & Stone (2003). The breeding structure of the British sheep industry, 2003. Defra 2006 (http://www.defra.gov.uk/evidence/economics/foodfarm/reports/documents/pollott2003.pdf). Accessed April 2010.

Sivam, S. K., Baylis, M., Gravenor, M. B. & Gubbins, S. (2006). Descriptive analysis of an anonymous postal survey of the occurrence of scrapie in Great Britain in 2002. *Vet Rec* **158**, 501–506. Medline

Truscott, J. E. & Ferguson, N. M. (2009). Control of scrapie in the UK sheep population. *Epidemiol Infect* **137**, 775–786, doi:10.1017/50950268808001064. Medline
